# Supplementary material for: Allogeneic Stem Cell Transplantation in Multiple Myeloma: Risk Factors and Outcomes in the Era of New Therapeutic Options—A Single-Center Experience
Source: Cancers (Basel). 2023 Dec 7;15(24):5738. doi: 10.3390/cancers15245738 (PMC10742138; doi:10.3390/cancers15245738)
Supplement: Supplementary file 1 [file cancers-15-05738-s001.zip › cancers-2722776-supplementary.pdf]

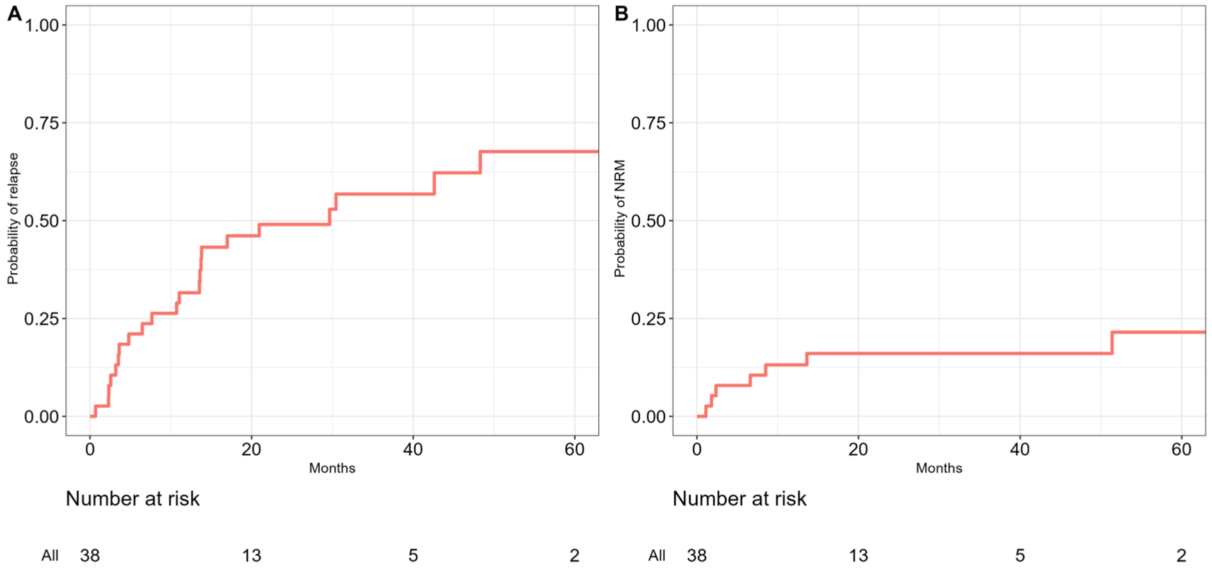

Supplementary Figure S1. Cumulative Incidence for Relapse and Non-relapse mortality, overall cohort
(A) Cumulative incidence for relapse. (B) Cumulative incidence for non-relapse mortality.

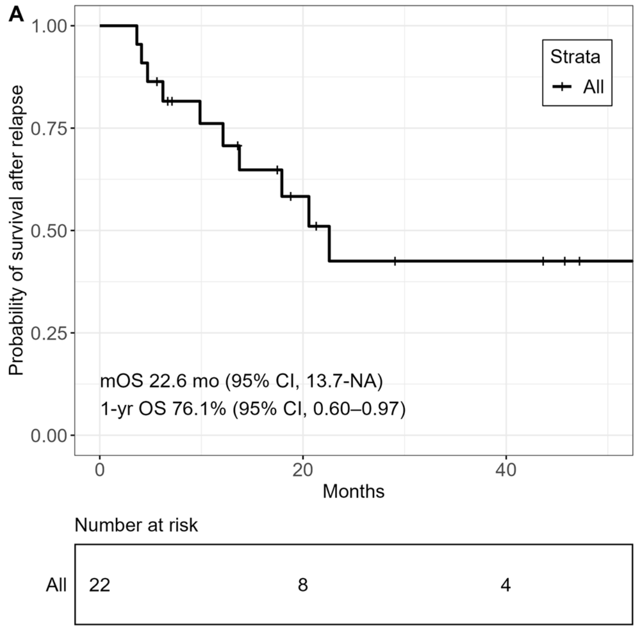

Supplementary Figure S2. Kaplan-Meier curve of overall survival from first relapse after allo-HSCT.

Supplementary Table S1. Number of therapy regimens and best response after relapse post allo-HSCT.

	2013-2018 (n=12)	2019-2022 (n=10)	All patients (n=22)
Median therapy regimens after relapse post allo-HSCT	6 (0-9)	4 (2-4)	4 (0-9)
Best response to subsequent treatment			
SCR	3 (25%)	3 (30%)	6 (27%)
CR	2 (17%)	1 (10%)	3 (14%)
VGPR	1 (8%)	1 (10%)	2 (9%)
PR	1 (8%)	1 (10%)	2 (9%)
< PR	4 (33%)	4 (40%)	8 (36%)
No subsequent treatment lines applied	1 (8%)	0 (0%)	1 (5%)

Allo-HSCT=allogeneic stem cell transplantation, CR=complete remission, LOT=line of therapy, PR=partial remission, SCR=stringent complete remission, VGPR=very good partial remission.

Supplementary Table S2. Therapies administered for relapse after allo-HSCT.

	**Relapsed patients** (n=22)
**Proteasome inhibitor**	17 (77%)
Bortezomib	3 (14%)
Carfilzomib	17 (77%)
Ixazomib	9 (41%)
**IMiD**	18 (82%)
Lenalidomide	8 (36%)
Pomalidomide	15 (68%)
**Monoclonal antibody**	17 (77%)
Daratumumab	11 (50%)
Isatuximab	4 (18%)
Elotuzumab	9 (41%)
**Chemotherapy**	9 (41%)
D-PACE	6 (27%)
DCEP	2 (9%)
Bendamustine	2 (9%)
Cyclophosphamide	1 (5%)
Venetoclax	5 (23%)
Selinexor	1 (5%)
Interferon	2 (9%)
Belantamab mafodotin	5 (23%)
Teclistamab	7 (32%)
Talquetamab	1 (5%)
Radiation therapy	5 (23%)
2nd allo-HSCT	1 (5%)

Allo-HSCT=allogeneic stem cell transplantation, IMiD=immunomodulatory drug, D-PACE=dexamethasone/ cisplatin/ doxorubicin/ cyclophosphamide/ etoposide, DCEP=dexamethasone/ cyclophosphamide/ etoposide/ cisplatin.
